# The Effects of Moderate Whole Grain Consumption on Fasting Glucose and Lipids, Gastrointestinal Symptoms, and Microbiota

**DOI:** 10.3390/nu9020173

**Published:** 2017-02-21

**Authors:** Danielle N. Cooper, Mary E. Kable, Maria L. Marco, Angela De Leon, Bret Rust, Julita E. Baker, William Horn, Dustin Burnett, Nancy L. Keim

**Affiliations:** 1 Department of Nutrition, University of California at Davis, 1 Shields Ave, Davis, CA 95616, USA; dncooper@ucdavis.edu (D.N.C.); adeleon@ucdavis.edu (A.D.L.); brust@ucdavis.edu (B.R.); jemadejska@ucdavis.edu (J.E.B.); 2 Western Human Nutrition Research Center, USDA-ARS, 430 West Health Sciences Drive, Davis, CA 95616, USA; Mary.Kable@ars.usda.gov (M.E.K.); William.Horn@ars.usda.gov (W.H.); Dustin.Burnett@ars.usda.gov (D.B.); 3 Food Science and Technology, University of California at Davis, 1 Shields Ave, Davis, CA 95616, USA; mmarco@ucdavis.edu

**Keywords:** whole grains, maize, brown rice, whole wheat, fasting glucose, fasting blood lipids, microbiota, bowel movement frequency, gastrointestinal symptoms

## Abstract

This study was designed to determine if providing wheat, corn, and rice as whole (WG) or refined grains (RG) under free-living conditions will change parameters of health over a six-week intervention in healthy, habitual non-WG consumers. Measurements of body composition, fecal microbiota, fasting blood glucose, total cholesterol, high density lipoprotein (HDL), low density lipoprotein (LDL), and triglycerides were made at baseline and post intervention. Subjects were given adequate servings of either WG or RG products based on their caloric need and asked to keep records of grain consumption, bowel movements, and GI symptoms weekly. After six weeks, subjects repeated baseline testing. Significant decreases in total, LDL, and non-HDL cholesterol were seen after the WG treatments but were not observed in the RG treatment. During Week 6, bowel movement frequency increased with increased WG consumption. No significant differences in microbiota were seen between baseline and post intervention, although, abundance of order *Erysipelotrichales* increased in RG subjects who ate more than 50% of the RG market basket products. Increasing consumption of WGs can alter parameters of health, but more research is needed to better elucidate the relationship between the amount consumed and the health-related outcome.

## 1. Introduction

Grains are a staple of the average American diet and therefore changes to grain products, especially the level of refinement, can have a notable effect on Americans’ consumption of fiber, minerals, and vitamins [1]. The topic of whole grains has come to wider public attention since the publication of the 2005 Dietary Guidelines for Americans, which recommends half of one’s daily grain intake (3–5 servings or 48–80 g for adults) should be in the form of whole grains [2]. However, there is still a huge disparity between the recommended level of whole grain consumption and the actual amount of consumption in the US with most adults only consuming on average 9.76 g of whole grains daily, as reported by the 2009–2010 National Health and Nutrition Examination Survey (NHANES) [3].

Botanically, grains are defined as the caryopsis or dried fruit (also called corn) of a cereal plant [4]. True cereals include wheat (*Triticum* spp.), corn/maize (*Zea mays*), rice (*Oryza* spp.), oats (*Avena* spp.), and barley (*Hordeum* spp.), whereas the pseudocereals include foods such as amaranth (*Amaranthus* spp.), buckwheat (*Fagopyrum esculentum*) and quinoa (*Chenopodium quinoa*) [5]. Grains are made up of three distinct components: the fibrous bran, the starchy endosperm, and the lipid containing germ. In addition to these three components, some grains, such as oats, grow within an inedible husk which is removed prior to human consumption. Whole grains are defined as the intact edible portion of the fruit of the cereal plant or the ground, cracked, flaked, or rolled fruit so long as the original proportions of the bran, endosperm, and germ are present in nearly the same proportions in the processed grain as were found in the intact grain [5,6].

Refined grains are grains that have been altered so that they are devoid of some, or all, of their naturally occurring germ and/or bran. In removing the germ, the shelf life of the grain is generally improved due to the absence of the lipid component of the germ that can become rancid [5]. Removal of the bran is often done to remove fibrous and potentially bitter components of the grain to improve the hedonic experience of the consumer and lighten the color of the resulting grain product [7]. Unfortunately, the removal of the bran and germ removes many bioactive components including vitamins, minerals, antioxidants, phenolics, flavonoids, carotenoids and critically fiber, particularly insoluble fiber [4,8]. To ameliorate some of the losses in the refining process and increase consumption of vitamins and minerals, enrichment or fortification is done with riboflavin, niacin, thiamin, folate, iron, and calcium, however nothing is done to increase the fiber level of refined grains [9].

In addition to not consuming enough whole grains, Americans are also not consuming enough fiber. The 2009–2010 NHANES data reported adults were only consuming on average 17 g of fiber per day despite the recommendation to consume 25 to 38 g per day [3]. Consumption of more whole grains has been suggested as an excellent way for Americans to bridge the fiber gap because many palatability studies have shown that in moderate proportions substituting whole grains for refined grains does not change the “liking” of grain based foods [3,10].

Consumption of whole grains is associated with decreased risk of type 2 diabetes and major chronic diseases, such as cardiovascular disease, and may even decrease the risk for some types of cancers, such as colorectal cancer [4,11,12]. Alteration of glucose homeostasis [13,14,15,16] and reduction of total cholesterol and low density lipoprotein (LDL) have been somewhat inconsistently reported in connection with increased whole grain consumption [12,13,17,18]. Beneficial alteration of the gut microbiota is often cited as a possible reason for health improvements seen with increased whole grain consumption, although a relationship between the two is not always observed [8,13,14,19]. Fiber can be mechanistically linked to all these health improvements and the content of fiber varies from grain to grain, which might explain some of the mixed reports. Another potential confounding variable is how much fiber subjects are consuming either prior to the dietary interventions or as part of their background diet [20,21].

Our study was conducted to document biological changes that occur with increased whole grain consumption. We hypothesized that compared to the subjects consuming refined grains the whole grain consumers would have lower concentrations of fasting blood glucose, total cholesterol and LDL, increased bowel movement frequency, a shift to midrange fecal firmness, and increased abundance of *Bacteroidetes*. The three grains most commonly consumed in the United States, wheat, maize, and rice [1] were used in the grain interventions, which were provided in weekly “market baskets” containing an array of either whole or refined grain foods. The number of servings per day provided to each subject was intended to be ~100% of the total grain recommendations for their maintenance calorie level.

We found that measures of fasting blood cholesterol significantly improved with the whole grain intervention. We also found that bowel movement frequency was significantly improved relative to percent consumption of the whole grains, and blood glucose tended to decrease as well with increased percent consumption of whole grains. Additionally, we observed increased abundance of order *Erysipelotrichales*, with greater amounts of refined grain consumption.

## 2. Materials and Methods

Prior to initiating the study, the study plan and consent form were reviewed by the Institutional Review Board (IRB) of the University of California-Davis and approved (IRB ID 235561). The study is registered with clinicaltrials.gov (NCT01403857).

### 2.1. Subjects and Study Design

This study was a six-week intervention trial that was preceded by a screening period to determine eligibility and a pre-intervention baseline test visit (Table 1).

Subjects were consented and went through a screening process to determine if they were eligible to participate based on inclusion and exclusion criteria. To be included, subjects needed to be healthy adults, between the ages of 19 to 46 years with body mass index (BMI) 20 to 28 kg/m^2^. They had to be “low whole grain consumers” consuming not more than 1 serving of whole grains/whole grain products per day, on average. This was evaluated using a screener that probed 81 different grain products including food items like breads, pastas, cereals, and snack bars. Subjects were asked to report how often they consumed these products by circling the option most appropriate to their consumption habits from consuming a serving of the product “never or less than once per month”, “1–3 per month” then one through seven times per week, to the greatest frequency queried “2 per day”. Subjects were disqualified for the study if they reported consuming seven or more servings of whole grain products per week. They also reported that their body weight had remained stable (within ±3 kg) for the past 6 months and they were not currently dieting to lose weight. It was also necessary that subjects be able to prepare and eat the majority of their meals at home.

Potential subjects were excluded if they reported having a diagnosis of type 1 or 2 diabetes mellitus, gastrointestinal diseases including malabsorption syndromes, chronic inflammatory bowel disease, colorectal cancer, celiac disease (gluten sensitivity), diverticulitis, Crohn’s disease; regular use of colonics and/or laxatives; recent (within 3 months) use of antibiotics, appetite suppressants, mood altering medications, and/or regular use of tobacco/tobacco products. Females were excluded if they were currently pregnant or were pregnant within the last six months. Eating habits were also queried, and subjects were excluded if the majority of meals were eaten away from home, in restaurants, or from fast food establishments. If qualified based on these criteria, a fasting blood sample was sent to a certified clinical chemistry lab at the University of California at Davis for a comprehensive clinical chemistry panel and a complete blood count to rule-out existing health problems, of which the subject may be unaware.

Eligible subjects were randomly assigned in blocks to the control (refined grain market basket) or treatment (whole grain market basket) groups to achieve a 1:2 ratio of those receiving refined grain to whole grain. Overall 46 subjects enrolled in the study. Results from the analysis of fasting blood samples are based on 45 subjects due to a missing post intervention sample; body composition data were available for only for 43 subjects due to equipment malfunction; gastrointestinal (GI) symptoms were assessed for 37 subjects due to incomplete record keeping by subjects, microbial analysis was performed on 28 subjects due to fecal sample loss or poor reading depth resulting from sequencing. Table 2 contains information about the subjects.

### 2.2. Market Baskets and Consumption Log

The market baskets consisted of foods made of either refined or whole wheat (representing ~75% of the products), corn (~15%), or rice (~10%). Three grain products were developed for the study: cookies, muffins and baking mixes; the others were commercially prepared items: bread, ready to eat cereals, couscous, crackers, pastas, rice, and tortillas (Table 3).

The contents of the market baskets (number of grain servings per week) were determined based on the caloric needs of the subjects, determined using the Harris-Benedict equation [22]. The calorie prescriptions were made in 200-kcal intervals. For example, a subject with an estimated energy expenditure of 1960 kcals would be provided a 2000 kcal per day basket and would receive six servings of grains per day, and a total of 42 servings of grains in their weekly market basket. All grain products were weighed prior to being given to the subjects. At the 2000 kcal level the whole grain market basket supplied 96 g of fiber, an average of 13.7 g per day whereas the refined grain market basket supplied 29.7 g of fiber, an average of 4.2 g per day. Subjects were asked to maintain their typical diet during the market basket intervention. We had planned to monitor dietary intake using unannounced multi-pass 24-h recalls collected by phone interview. However, we were unable to obtain a sufficient number of recalls, and between-interviewer variation was problematic. Thus, we have deemed the 24-h recall data of poor quality and have chosen not to present those data.

**Figure A1:**
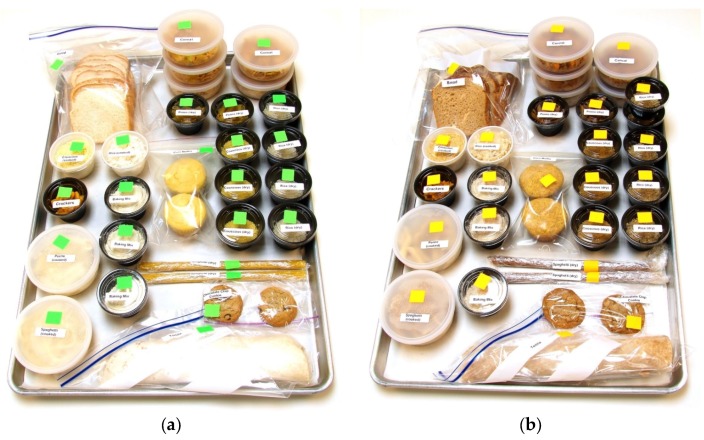
Images of the 2000 kcal market baskets: (**a**) the refined grain basket; and (**b**) the whole grain basket.

Subjects were asked to record the type, amount, preparation, as well as date and time of the grains used from the market basket. The grain products were pre-portioned for the subjects to increase convenience and help with record keeping in the log books (Figure A1). Subjects were not required to consume all grain products but where encouraged to replace what they would normally consume with products from the provided market baskets whenever possible. The log data were compared with the disappearance data generated by weighing back the returned market basket containing the prepared or unprepared unused grain products. If there was a discrepancy between the log data and what was returned in the market basket, in absence of a note from the subject explaining the discrepancy, the weigh back data was deemed preferred and used in the consumption calculation. After the initial basket was picked up subjects returned once weekly to return their past week’s unused food, return their log books, and pick up materials for the following week. The intervention period was six weeks. The post-intervention test day was scheduled during the sixth week.

### 2.3. Body Composition

Subject’s body composition was determined using air displacement plethysmometry (BodPod, COSMED, USA, Inc., Concord, CA, USA) in conjunction with a calibrated digital scale (Scale-tronic model 6002, Wheaton, IL, USA). Height was determined by a wall-mounted stadiometer (Ayrton Stadiometer model S100, Prior Lake, MN, USA). Subjects were required to wear tight fitting clothing such as a bathing suits or compression shorts as well as swim caps prior to entering the chamber. Subjects were evaluated twice, once in the baseline period and once after the sixth week of intervention, on both occasions they were tested after an overnight fast.

### 2.4. Clinical Parameters

Twelve-hour fasting blood samples were obtained by a licensed phlebotomist twice during the study. Whole blood was sent to the UC Davis Medical Center’s clinical laboratory for analysis of glucose, lipids, cell counts, and iron status. The first collection occurred at baseline, the second occurred post intervention.

### 2.5. Gastrointestinal Symptoms Log Book

GI symptom log books were distributed and collected weekly with the market baskets. The goal of the logs was to monitor GI tolerability of the market basket products. Subjects were asked to record the day and time of each bowel movement and rate the consistency of each bowel movement using the Bristol stool scale [23]. There was also a short questionnaire in the log book that asked subjects to reflect over the past week when reporting their answers. The questions regarded frequency of experiencing gas, bloating, abdominal pain, nausea, or flatulence, and responses were recorded on a 5-point Likert scale [24]. There was a query about experiencing a change in stool and, if so, how they felt about the change.

### 2.6. Fecal Collection

A single bowel movement was collected by the subject at baseline and then again post intervention using a feces collection kit. The kit consisted of a plastic container lined with a ziplock bag, gloves, pens for labeling, and a hard-sided cooler with dry ice to keep their samples frozen until they could be delivered to the research center (WHNRC) for immediate storage at −20 °C.

### 2.7. Gut Microbial Community Analysis

The composition of the fecal microbiota was determined by sequencing of bacterial 16S rRNA genes. Comparisons of the fecal microbiota at baseline and post intervention were used to determine the change in relative abundance of specific taxa. Bacterial DNA was extracted as previously described [25]. Briefly, approximately 200 mg of fecal material was placed in a 2 mL screw cap tube containing 300 mg of 0.1 mm diameter zirconia/silica beads (Biospec Products, Bartlesville, OK, USA), the mixture was treated with lysozyme from the QIAamp DNA Stool Mini Kit (QIAGEN) and held for 30 min at 37 °C. Next mechanical lysis was performed by bead beating for 1 min, twice, at 6.5 m/s (FastPrep-24, BP Biomedicals, Santa Ana, CA, USA) in 1.5 mL ASL buffer [25,26]. Finally, the suspension was heated to 95 °C for 5 min while shaking at 500 rpm. DNA was then purified using the QIAamp DNA Stool Mini Kit (QIAGEN) according to the manufacturer’s instructions.

The V4 region of the 16S rRNA gene was selected for PCR amplification because it has been shown to faithfully represent the taxonomic profile of microbial communities relative to characterization of the full length 16S gene sequences [27]. Primers F515 and R806 [28] were used to amplify the 16S rRNA V4 region from each purified DNA sample. An eight base pair (bp) barcode was present on the 5’ end of primer F515 [29] to facilitate demultiplexing of pooled sequence samples during downstream analysis. The PCR products were pooled and gel purified using the Wizard SV Gel and PCR Clean-Up System (Promega, Madison, WI, USA). Amplicons were then sent to the UC Davis Genome Center (http://www.genomecenter.ucdavis.edu/) for library preparation and paired-end 250 bp sequencing using the Illumina MiSeq platform (San Diego, CA, USA).

QIIME (Quantitative Insights into Microbial Ecology) [30] was used to join paired ends [31], quality filter, and demultiplex the sequencing data. Chimeras were identified using USEARCH [32,33] and removed. Operational Taxonomic Units (OTUs) were picked from the assembled sequences using the open reference OTU picking method and a threshold of 97% pairwise identity [30]. Very low abundance (0.005% or less) OTUs were removed prior to statistical analysis [34].

### 2.8. Statistical Methods

Microsoft Excel (Microsoft, Redmond, WA, USA)) was used to format data. JMP (JMP^®^, Version 12.1.0. SAS Institute Inc., Cary, NC, USA) and R (R, Version 3.3.2, R Foundation for Statistical Computing, Vienna, Austria) were used for analysis. Data were tested for normal distribution using the Shapiro–Wilks test of normality and if non-normally distributed transformation using Box Cox was performed, if transformation was unsuccessful (*w* above 0.96) nonparametric testing was used. Body composition data and fasting blood data were analyzed using linear regression and analysis of variance using baseline measures as a cofactor in the regression model. Gastrointestinal symptoms, bowel movement frequency, Bristol scores of feces, and microbial abundance could not be normalized using Box Cox transformations so non-parametric Wilcoxon Signed Ranks tests were used to determine significance following logistical regression [35]. For the microbial data, OTU counts were rarefied to a depth of 15,000 sequences per sample prior to analysis. This number was chosen because at this sequencing depth the number of unique OTUs observed was no longer exponentially increasing. Samples from two subjects on the whole grain market basket intervention had fewer than 15,000 sequences and so were discarded prior to analysis. Taxa that were present in at least 2% relative abundance in at least one sample were analyzed for differential abundance between experimental groups using LefSe [36]. For the purposes of determining fold change, sequence counts of 0, when they occurred, were replaced with a sequence count of 1. Due to the increased risk of type 1 errors with multiple comparisons, the Benjamini–Hochberg False Discovery Rate Procedure was implemented to reduce the risk of false discovery [37].

## 3. Results

### 3.1. Market Basket Consumption

**Figure 1:**
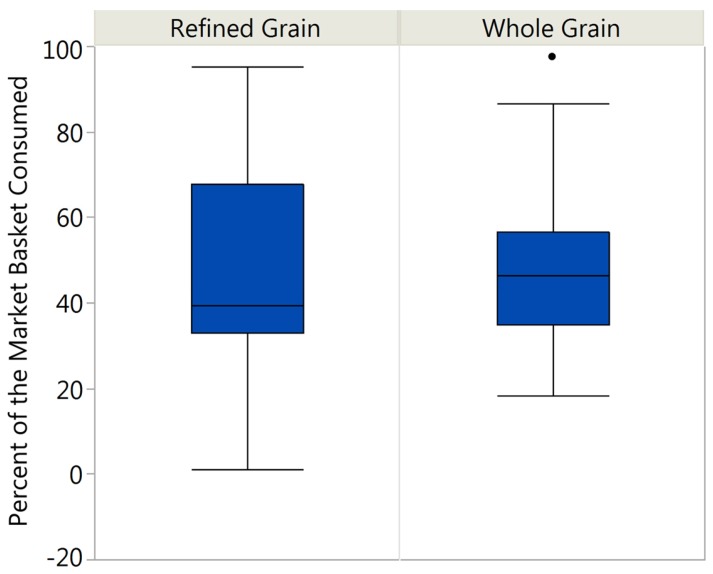
Range of Market Basket Consumption. Consumption of the refined grain market basket ranged from 1.1% to 95.1% with the average consumption being 44.7% and a standard error of the mean of 7.8. Consumption of the whole grain market basket ranged from 18.1% to 97.5% with the average consumption being 47.9% and a standard error of the mean of 3.0.

There was a wide range of consumption of the market baskets products with refined grain consumption ranging from 1.1% to 95.1% and whole grain consumption ranging from 18.1% to 97.5% (Figure 1).

### 3.2. Changes in Body Composition from Baseline to Post-Intervention

**Figure 2:**
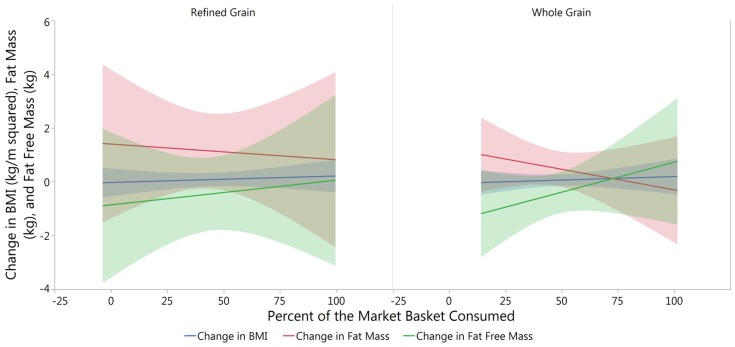
Changes in Body Composition with Percent Consumption of the Market Baskets. No significant differences were found using analysis of variance in the change of BMI (RG *p* = 0.494, WG *p* = 0.658), fat mass (RG *p* = 0.962, WG *p* = 0.372), or fat free mass (RG *p* = 0.823, WG *p* = 0.561) from baseline to post intervention.

Air displacement plethysmometry performed at baseline and post intervention was used to calculate the change in Body Mass Index (BMI), fat mass, and fat free mass between baseline and post intervention. Using baseline measures as a cofactor linear regression followed by analysis of variance were performed in the treatment groups and no significant differences were seen (Table 4). Similar analysis was performed on each treatment group relative to the percent of the market basket consumed and significant differences were still not observed (Figure 2).

### 3.3. Changes in Fasting Blood Glucose from Baseline to Post-Intervention

**Figure 3:**
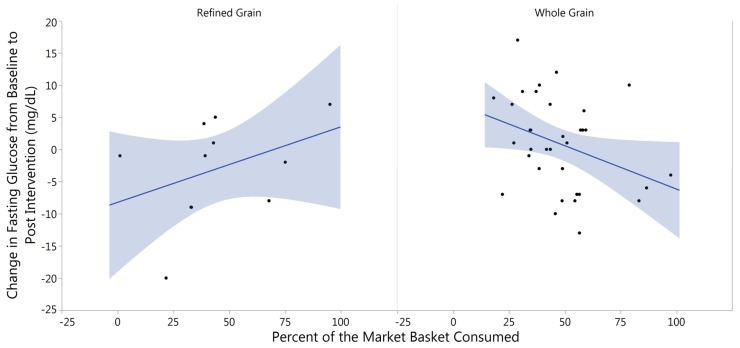
Percent of Market Basket Consumed Related to Change in Fasting Glucose. Increased consumption of the whole grain market basket was related to lower (*p* = 0.053) fasting blood glucose in the whole grain treatment, whereas there was a pattern of increased blood glucose with increased consumption of the refined grain market basket (*p* = 0.590).

Similar to our findings with body composition, we found there was no significant impact of the type of market basket consumed on fasting blood glucose and the mean change for both treatment groups was negligible (RG = −3.00 ± 2.38 mg/dL; WG = −0.29 ± 1.62 mg/dL; *p* = 0.250). However, the percent of the WG market basket consumed trended with decreased fasting blood glucose when looking at the change in blood glucose from baseline to post intervention while controlling for baseline levels using linear regressions followed by analysis of variance (*p* = 0.053). This observation was not replicated with RG market basket consumption (*p* = 0.590), indicating that a certain quantity of whole grain consumption may be reducing fasting blood glucose (Figure 3).

### 3.4. Changes in Fasting Blood Lipids from Baseline to Post Intervention

**Figure 4:**
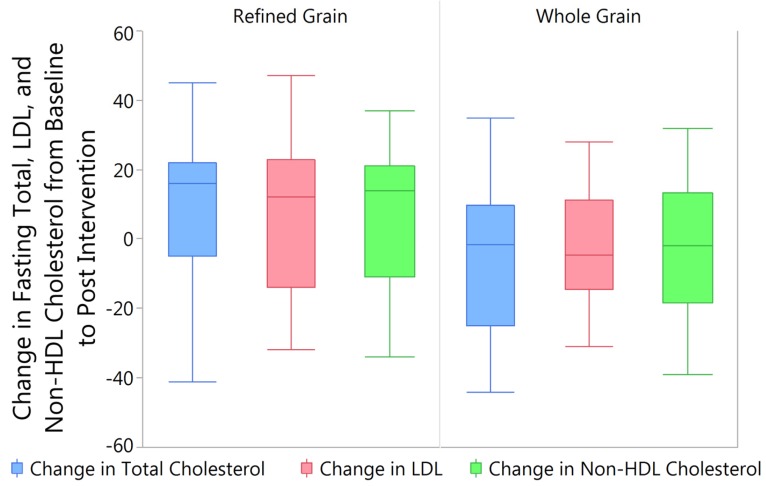
Consumption of the Whole or Refined Grain Market Basket Related to Change in Fasting Total, LDL, and Non-HDL Cholesterol. Consumption of the WG market basket was significantly associated with lower fasting levels of total cholesterol (*p* = 0.018), LDL cholesterol (*p* = 0.035), and non-HDL cholesterol (*p* = 0.047) when compared to subjects that consumed the RG market basket.

When observing the changes in fasting blood lipids from baseline to post intervention between the RG and WG treatments, while controlling for baseline levels, significant differences were seen in total cholesterol (*p* = 0.018), LDL cholesterol (*p* = 0.035), and in non-high density lipoprotein (HDL) cholesterol (*p* = 0.047) (Figure 4). Negligible changes were seen in HDL cholesterol (RG = 1.55 ± 1.49 mg/dL; WG = −2.41 ± 1.31 mg/dL; *p* = 0.178) and triglycerides (RG = 2.73 ± 14.47 mg/dL; WG = 6.29 ± 8.97 mg/dL; *p* = 0.799).

When comparing fasting lipid levels to percent consumption of the market baskets while controlling for baseline levels no significant differences were seen in the change in total (RG *p* = 0.179; WG *p* = 0.122), LDL (RG *p* = 0.0682; WG *p* = 0.265), HDL (RG *p* = 0.972; WG *p* = 0.816), or non-HDL (RG *p* = 0.313; WG *p* = 0.313) cholesterol, or triglycerides (RG *p* = 0.790; WG *p* = 0.313).

### 3.5. Gastrointestinal Tolerability

Gastrointestinal symptoms were reported by subjects in their weekly log books. Table 5 represents the self-reported gastrointestinal symptoms of bloating or gas, abdominal pain, nausea, flatulence, changes in stool, change in feelings about changes in stool, bowel movement frequency, and Bristol stool score for weeks one and six during the market basket intervention.

**Figure 5:**
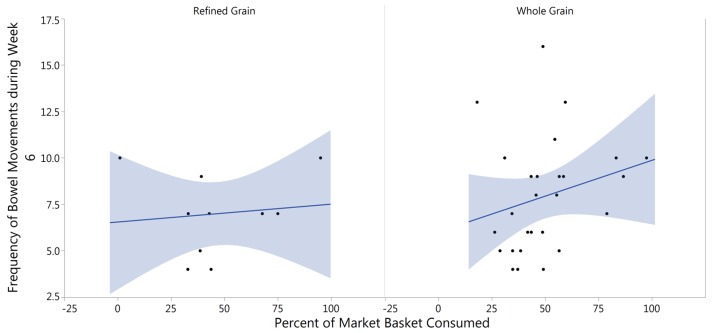
Percent of market basket consumed related to bowel movement frequency. The association between bowel movement frequency and percent of market basket consumed for refined grain consumers (**left** panel) and whole grain consumers (**right** panel) as determined by logistical regression and Wilcoxon Rank Sum testing. The refined grain treatment represents data from ten subjects; the whole grain treatment represents data from 27 subjects. There is a significant positive association with whole grain consumption (*p* = 0.046), but not with refined grain consumption (*p* = 0.407).

The average bowel movement frequency for subjects during the sixth week of the intervention was compared to the average grain product consumption (percent consumed) by rank (50% or higher consumption verses less than 50% consumption) by subject in each treatment group, respectively. Using logistical regression and Wilcoxon Rank Sum testing, it was determined that there was a significant increase in bowel movement frequency with increased consumption of the whole grain market basket (*p* = 0.046). There was no association between increased frequency and the refined grain treatment (*p* = 0.407) (Figure 5).

### 3.6. Fecal Microbiota Analysis

To determine if there were significant changes in the gut microbial community mediated by diet, which could then potentially influence changes in the health parameters observed, we sequenced the 16S rRNA V4 region of the bacterial DNA in subject stool collected at baseline and after six weeks of either WG or RG market basket consumption. A total of 2,983,060 sequences were obtained after quality filtering with an average of 48,100 sequences per sample. Two subjects in the WG market basket treatment were excluded from the microbiota analysis because one each of their samples was not sequenced sufficiently to effectively represent the overall community structure.

**Figure 6:**
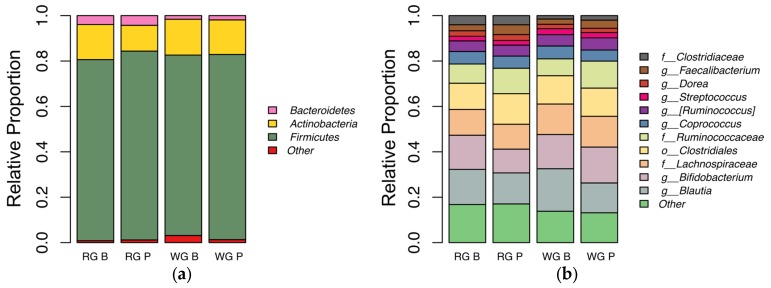
Relative proportion of taxa in each experimental group. The relative proportion of taxa at baseline (B) and post intervention (P) for the refined grain (RG) and whole (WG) treatments: (**a**) the relative proportion of bacteria at the phylum level; and (**b**) the relative abundance of bacteria at the most specific level of classification available. In both panels, taxa present at a median of 1% relative abundance in the data set are shown. Taxa present at lower than 1% median relative abundance are grouped into the “Other” category.

Overall, *Firmicutes* was the most relatively abundant phylum detected in our subjects, as has previously been observed in studies examining urban adult human gut microbiota using stool samples [38,39]. The second most abundant phylum was *Actinobacteria* with *Bacteroidetes* coming in third. The high proportion of *Actinobacteria* in our samples was driven primarily by a high representation of the genus *Bifidobacterium*, which composed as much as 40% of one sample prior to whole grain market basket consumption and was present at a median 10% and 16% relative abundance at baseline prior to WG and RG market basket consumption, respectively. Change in abundance during market basket consumption was not significant and this might be explained by the high levels present prior to consumption (Figure 6).

**Figure 7:**
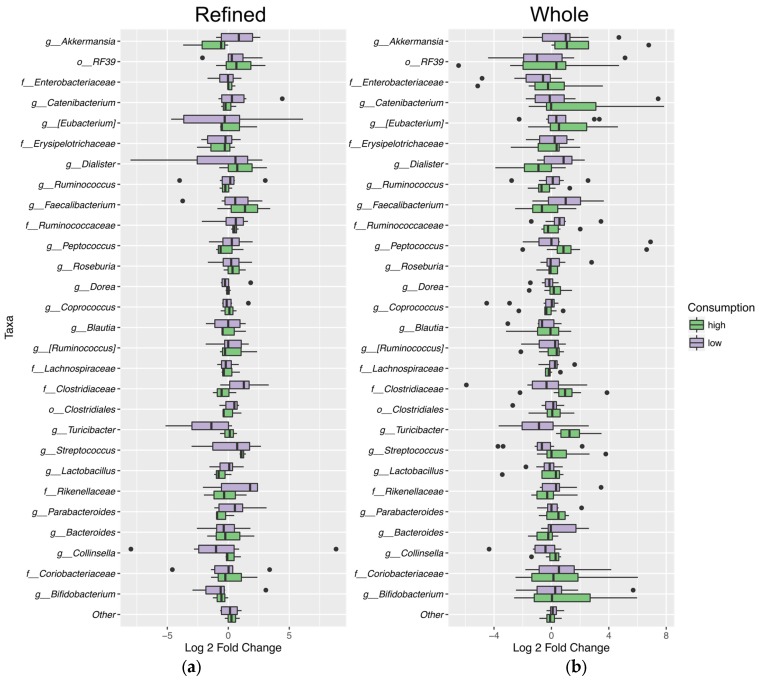
Variation in specific taxa during market basket consumption. Log2 fold change in abundance of bacteria at either high (50% or more) or low (49.99% or less) levels of consumption of the market baskets from baseline to post intervention for: refined grain (**a**); and whole grain (**b**). Taxa shown were present in at least 2% relative abundance in at least one sample. Dots seen in the graphs represent outliers. The findings of interest were an increase in the relative abundance of *Akkermansia* and *Lactobacillus* with high whole grain market basket consumption and a decrease with high refined grain consumption, as well as the increased abundance of the order *Erysipelotrichales* (*p* = 0.023) with high refined grain consumption.

Although there was no significant difference in the relative abundance of any particular taxa between experimental groups, six of eight individuals consuming a high proportion (50% or greater) of the WG market basket showed increased relative abundance of *Akkermansia* and *Lactobacillus*, while two of three individuals consuming a high proportion of the RG market basket showed decreased relative abundance of this organism. There was also a trend for increased abundance of order *Erysipelotrichales* (*p* = 0.023) with high (50% or greater) RG market basket consumption. In our dataset, order *Erysipelotrichales* included unidentified members of family *Erysipelotrichaceae* and the genera *Eubacterium* and *Catenibacterium* (Figure 7).

## 4. Discussion

Consumption of the whole grain market basket products for six weeks was associated with a significant decrease in total, LDL, and non-HDL cholesterol compared to subjects consuming the refined grain market basket. Health parameters including fasting glucose, and bowel movement frequency were associated with a higher percentage of consumption of the whole grain market basket. No significant differences were seen when comparing the change from baseline to post intervention between treatment groups for the following parameters: BMI, fat mass, fat free mass, glucose, HDL, triglycerides, gas or bloating, abdominal pain, nausea, self-reported change in stool, feelings about changes in stool, bowel movement frequency, Bristol stool score, or abundance of microbiota.

### 4.1. Fiber Intake

Market baskets were designed to mimic the types of grain products typically consumed by Americans as closely as possible, which is why the baskets featured a majority of wheat products (~75%), some maize products (~15%), and rice (~10%), since those proportions are similar to the national availability of those grains as reported by USDA Economic Research Service [1]. The whole and the refined grain market baskets were matched as closely as possible in terms of types of foods provided, but the difference in the amount fiber between market baskets was substantial. At the 2000 kcal level, the whole grain market basket supplied 96 g of fiber, an average of 13.7 g per day, whereas the refined grain market basket supplied only 29.7 g of fiber, an average of 4.2 g per day, if all of the products were consumed. The average consumption of the market basket in this study, when rounded, was 47 percent. Thus, subjects in the refined grain group were consuming only about 2 g per day of fiber from the provided grain products whereas the whole grain subjects were consuming about 7 g per day of fiber from the grain products. Even though this is an average of a five-gram difference in fiber consumption between the grain treatments, whole grains at this percent consumption are not contributing a significant amount of fiber to the daily diet. Fibers such as beta glucan have been shown to reduce cholesterol and LDL with as small a dose as three grams per day [40]. However, the major sources of fiber in wheat are arabinoxylans, hemicellulose, and only very small amounts of beta glucan [41]; corn fibers are hemicellulose, arabinose, xylan, lignin, and resistant starch [42]; and rice contains cellulose, pectic fibers (arabinans, arabinogalactans, and galacturonans) and hemicellulose [21,43,44,45,46]. Not much is known about the dietary impacts of these individual fibers. Since background diet was not evaluated during the intervention it is possible that subjects in the whole and refined grain treatment groups were consuming vegetable and or fruit fiber at different levels from each other. Assuming we were successful at disqualifying habitual medium or high whole grain consumers, we were confident that the enrolled subjects were not consuming a large amount of cereal fiber outside of the intervention. Not having a reliable estimate of the subject’s fiber intake outside of the intervention limits any conclusions we can draw about fiber intake during the study, and how the difference in fiber provided by the market baskets may have influenced the outcome of individual subjects.

### 4.2. Fasting Glucose

When comparisons were made taking into account what percentage of the market basket was consumed, a nearly significant trend was observed: as whole grain consumption increased fasting glucose levels decreased. A potential mechanism behind this theory is that due to the increased fiber of whole grains they remain as larger particles post mastication and so potentially are more challenging to mechanically and enzymatically degrade resulting in lower, longer releases of glucose into the blood stream and subsequently a more gradual secretion of insulin. However, this effect may not explain why a decrease in glucose might exist after something as long as a 12-h overnight fast [47,48,49,50]. Another potential mechanism that may explain the persistence of decreased glucose levels with increased consumption of whole grains, even after an overnight fast, is that the increase in fiber from whole grains may alter metabolism in the microbiota increasing the production of short chain fatty acids (SCFA) like propionate and acetate due to continued exposure to fiber from the whole grains. Propionate is known to stimulate enteroendocrine L cells to increase the release of glucagon-like peptide-1 (GLP-1) which increases the responsiveness of insulin to glucose as well as inhibiting gastric emptying [51,52,53]. In a human study, supplementation with propionate was shown to decrease glucose peaks and two-hour glucose area under the curve [54]. Acetate may be able to alter appetite and satiety signaling in the brain without use of gut hormone intermediaries, and has been shown to reduce fasting glucose levels in humans [55,56]. Reduced blood glucose has been seen postprandially in the literature with boiled barley, whole rye, whole wheat, and brown rice in comparison to refined grain interventions [46,57,58]. Fasting glucose has been shown to decrease with acute interventions of wheat bran in mice [50] and with acute interventions of whole wheat, whole oats, and whole barley in rats [59]. There is some literature suggesting that whole grains do not have an effect on glycemic control. For example Ampatzoglou and colleagues showed no significant impact of increasing whole grain consumption from 24 or more grams per day to at least 80 g per day for six weeks, on blood glucose [13]. Another study by Kristensen and colleagues compared acute whole grain wheat and refined grain wheat consumption and failed to show significant differences in blood glucose up to 180 min after feeding [60]. Since only a trend towards decreased glucose levels was observed in our study no strong conclusions can be drawn, but it is clear that future research will be needed to definitively understand if beginning or increasing whole grain consumption may effect glycemic control, and if so why.

### 4.3. Blood Lipids

Fasting total, LDL, and non-HDL cholesterol decreased with consumption of the whole grain market basket, whereas no significant changes were seen with these lipids in the refined grain group. Whole grain consumption has been shown to improve lipid profiles [12]. In rats diets high in maize have been shown to reduce LDL [61]. In humans diets rich in whole wheat have been shown to reduce total cholesterol when given at a dosage of 48 g per day [15,19]. Most of the research on the lipid lowering effects of whole grains has been performed with oats, which are rich in beta glucan, a fiber that has been clinically shown to lower cholesterol [40]. Oats were not featured in this study and while whole wheat contains very small amounts of beta glucan, maize and rice do not [21,41]. The current understanding of this lipid lowering property of whole grains is that it is due to their increased fiber content as compared to their refined grain counterparts [8,41].

Assuming whole grain intake replaces refined grain intake this diet alteration would be expected to increase fiber intake, thereby initiating the following signaling cascade. Fiber increases the viscosity of the foodstuffs in the stomach which may delay gastric emptying and affect satiety signaling. In the duodenum the increased viscosity may also alter nutrient release from the chyme and thus affect nutrient sensing, which could have far reaching consequences including lipid metabolism and processing [62]. The better established and accepted mechanism for increased fiber intake decreasing blood cholesterol levels is through sequestration of bile salts resulting in their loss in feces instead of their reabsorption back into the intestine, which results in the body needing to use cholesterol to manufacture more bile salts, thus decreasing blood cholesterol concentrations [58,63]. The existing literature suggests that in some cases increasing whole grains consumption does not affect lipid levels. This was seen in the previously discussed mixed increased whole grain consumption study by Ampatzoglou and colleagues [13]. Odes and colleagues also performed a study on humans using a mixed grain fiber supplement providing 12.5 g of fiber daily for two or four weeks and found that it had no effect on HDL or LDL cholesterol [64]. However, the findings of the current study indicate that inclusion of a combination of whole grain wheat, corn and rice in the diet for six weeks led to decreased fasting total, LDL, and non-HDL cholesterol levels compared to consuming the refined grain counterparts.

### 4.4. Fecal Frequency

While there was no difference in average bowel movement frequency across the six-week intervention, there was there a significant difference in frequency with relation to percent of the whole grain market basket consumed when looking at only the sixth week of intervention. Bowel movement frequency increased significantly with increased whole grain consumption in Week 6 while there was only a slight, non-significant increase in the refined grain intervention. Whole grains have been shown to decrease intestinal transit time, thereby increasing bowel movement frequency [8,41,65,66]. In a recent review of 65 intervention studies, it was found that intake of wheat fiber, such as is found in wheat bran, a component of whole grain wheat, was shown to decrease transit time by about 45 min per gram of wheat fiber consumed, if the initial transit time was more than 48 h [67]. In a meta-analysis of 65 intervention studies utilizing cereal fibers from wheat, rye, corn, oats, barley, sorghum, and rice, investigators reported that, if the initial transit time was more than 48 h, cereal fiber reduced transit time by 30 min per gram consumed [68]. The mechanism for this is generally thought to be fecal bulking due to the increased fiber from increasing whole grain intake causing water retention in the feces which improves transit time [8,69]. Consuming whole grain barley has been shown to increase average fecal weight compared to the stool weight of refined grain consumers [65] and consumption of a high fiber cereal made of wheat, corn, oats, and soybeans was shown to increase bowel movement frequency compared to a similar low fiber cereal in humans [70]. It must be noted that bowel movement frequency was self-reported in our study and was not measured pre-intervention at baseline. There was also a trend towards increased bowel movement frequency observed in the refined grain intervention, thus the validity of the increase should be interpreted with caution.

### 4.5. Microbiota

Increasing intake of whole grain wheat, maize, and barley have all been shown to alter the human gut microbiota, potentially through increasing the availability of fiber, but possibly due to other functional components in the whole grains such as polyphenols [14].

However, in this study no significant changes in microbial community composition were detected with consumption of the whole or refined grain market baskets. This is not necessarily surprising, given that in this pilot study subjects were free-living and the market basket comprised only about 16% of their expected calorie intake at the average level of consumption.

Although no significant differences were detected between the whole and refined grain treatments this does not empirically mean that there were not changes in the microbial community that were simply unable to reach statistical significance with our small sample size. Additionally, it is possible that changes occurred in the metabolic pathways or activity of the microbiota over the course of this investigation that could be detected with metatranscriptomic, metabolomics, or proteomic analysis [71]. For example, changes in the production of metabolic byproducts such as short chain fatty acids can occur without necessitating changes in bacterial composition [72,73,74]. Alterations in bacterial metabolism can occur due to changes in pH, oxygen tension, or substrate availability, to name a few [75].

Based on studies seen in the literature it was expected that the increase in dietary fiber from the consumption of 50% of more of the whole grain market basket would initiate an increase in the abundance in *Bifidobacterium* [19,76,77,78]. At baseline, high levels of *Actinobacteria* were seen, largely due to an enrichment of *Bifidobacterium.* Enrichment of *Actinobacteria* has previously been observed in expectant mothers in the third trimester of pregnancy [79], however since subjects were excluded if pregnant it is unclear to us why *Actinobacteria* was so abundant at baseline. It is possible that subjects were consuming a significant amount of dietary fiber from a non-whole grain source prior to the study, which could have selectively enriched *Bifidobacterium* [80]. If this were the case, background diet variability may have acted as a confounding factor obscuring the expected increase in the abundance of *Bifidobacterium*. The average fiber intake for American adults is 17.0 g per day with grain products as well as mixed dishes (containing grains) make up nearly half (46%) of the intake, whereas vegetables account for 16%, snacks and sweets for 13% and fruits 12% [81].

The trend towards increased relative abundance of *Akkermansia* and *Lactobacillus* with high whole grain consumption seen in this study has been previously observed with a whole grain barley feeding study in rats [82]. Increases in the abundance of *Lactobacillus* have also been seen in humans after a diet rich in whole grain barley [83] and whole grain wheat [19]. While this trend was not significant, we feel it is a noteworthy observation since increases in *Akkermansia* are associated with reduced endotoxemia, improved inflammatory tone, and potentially weight loss [84,85,86,87]. *Lactobacillus* are often taken as probiotics due to their ability to exclude pathogenic bacteria and prevent or shorten episodes of diarrhea [88].

An increase in the abundance of the order *Erysipelotrichales* was seen in this study in the three individuals that consumed 50% or more of the refined grain market basket. Increased abundance of *Erysipelotrichales* has also been reported in canines with a diet high in refined maize and low in fiber [89], in mice with high fat diets [90], in mouse models of acute inflammatory colitis [91], and in humans with Crohn’s disease [92]. Within the order *Erysipelotrichales*, the genera *Eubacterium* and *Catenibacterium* as well as unidentified members of family *Erysipelotrichaceae* were observed, although no significant differences were detected at the family or genus levels. *Eubacterium* has been seen in lower abundance in individuals with metabolic syndrome [93] and advanced colorectal adenoma [94]. *Catenibacterium* has been observed to be enriched in individuals with end stage renal disease [95] and to be depleted in individuals with higher risk for cardiovascular disease [96]. *Erysipelotrichaceae* has been observed as being enriched with obesity, Western-type diets, and increased host cholesterol metabolites [97].

### 4.6. Limitations

A serious limitation of the study was the small sample size. The study enrolled 46 subjects but due to technical problems with instrumentation and only partial cooperation of some of our research volunteers, we have missing data for some variables reported here. Most notably fecal samples from only 28 subjects were available for microbiota analysis. Of those 28 subjects used for the microbiota analysis the ratio of sexes was not balanced. There was a 1:2 ratio of females to males in the refined grain treatment and about a 3:1 ratio of females to males in the whole grain treatment. When observing gut microbiota there is enormous variation between subjects, and within subjects, so it is difficult to detect the signal above the noise, as it were, especially with a small population of subjects. Only having one baseline and one post intervention blood draw and fecal collection was another limitation. Much of the data were collected through self-report which introduces its own complexities and errors, especially when it concerns dietary record keeping, however pre-weighing and re-weighing the market baskets was used to improve the accuracy of those data. Background diet was attempted to be assessed with phone interviews using the multi-pass 24 hour recall method, however subject cooperation was extremely poor so the data collected were not considered to be representative of the usual diet intake. Due to this limitation, we cannot say with certainty that subjects did maintain their habitual diet, creating another possible source of error. Estimated fiber intake from the market basket products was calculated, not determined directly by analysis, so it is possible that the quantity of grain fiber consumed was erroneous. However, since those values were then averaged across the week and by all subjects in the treatment group, this might improve the reliability of the estimations. Analysis was done for body composition, blood work, and GI symptoms using linear regression which can only be used to discover medium to large effects with a sample size as small as those utilized in this study; small changes many have occurred but would not have been detectable. This limits the ability to conclude with certainty that changes did not occur if no difference was detected in our analysis.

## 5. Conclusions

The results of this paper indicate that increasing the consumption of whole grains in habitual low-whole grain consumers may significantly lower fasting measures of total, LDL, and non-HDL cholesterol.

The trend towards decreased fasting glucose with increased whole grain consumption came tantalizingly close to significance, and was not seen when the same analysis was done with those receiving the refined grain intervention. Due to the tight regulation of blood glucose in healthy individuals, it is not surprising that changes to fasting levels would be subtle and challenging to detect without large groups of homogenous subjects. The fact that a strong trend was seen indicates that a true effect may be present and detectable with better study design.

The increase in bowel movement frequency seen with increased consumption of whole grains during the sixth week of the intervention is supported by existing literature and meta-analyses, however given that no baseline measurements were taken, the sample size was small (*n* = 37), the data rely solely on self-report, and that the same pattern was seen in the refined grain intervention this finding must be interpreted with some reservations.

Microbial analysis was conducted on 28 subjects, and we did not have the power needed to detect anything but very large changes in the microbial community. The only significant change seen was an increase in abundance of the order *Erysipelotrichales* in the three individuals that consumed 50% or more of the refined grain market basket. This finding cannot reasonably be interpreted to say that other changes to the microbiota were not occurring, since there was not enough power in the analysis to validate that inference. The microbial analysis performed in this study was exploratory and should not be interpreted without the many caveats discussed above.

## 6. Future Research

Given the small sample size and limited sample collection, future research would be necessary to definitively describe changes in health parameters seen with increased whole grain consumption. This study does indicate that whole grains may be an important food type for improving fasting cholesterol and glucose levels, as well as increasing bowel movement frequency. Due to the fact that some changes in health parameters only became apparent in subjects who consumed at least 50% of the whole grain market basket, another direction for future research would be to determine what level of whole grain consumption affords the most beneficial health effects. Due to differences in the composition and level of fibers, vitamins, minerals, and digestibility of different grains, it would also be important to consider exactly which whole grains are being studied and whether they are similar to the grains, and preparations of grains, seen in the population relevant to the study. Another possible direction for future research would be to compare the whole grains typically consumed in the US (wheat, corn, and rice) to other sources of whole grains such as oats, barley, and rye for which more definitive health effects have been documented. Then, recommendations could be further refined to include not only the total quantity of whole grains, but also more specifically for the types of grains consumed based on the potential health benefit.

It is likely that the improvements seen from increasing whole grain consumption come from the increases in fiber consumption, but future research is necessary to confirm this assertion and determine if other functional components of the whole grains contribute to health benefits. Regardless of how whole grains exert their effects, increasing whole grain consumption can reduce the gap between the recommended consumption of fiber and current fiber intake in the US.

## Figures and Tables

**Table 1:** Study design and procedures.

Screening	Baseline Test Day	Six Week Intervention (Each Event Occurred Weekly)	Post-Intervention Test Day
Consent to participate Determine eligibility	Fasted blood draw Fecal sample collected Body composition testing	Pick up the market basket and dropping off the last week’s unconsumed food Complete and return logs of: market basket consumption bowel movements gastrointestinal symptoms	Fasted blood draw Fecal sample collected Body composition testing

**Table 2:** Demographic information of subjects who received refined grain (RG) or whole grain (WG) market baskets ^1^.

Subjects	Sex	Age (Years)	BMI (kg/m^2^)	Calculated Daily Calorie Needs (kcal/day)	Percent of Market Basket Consumed
	F = Female				
	M = Male				
**All Subjects in Sample**					
Total (*n* = 46)	25 F, 21 M	25.8 ± 0.9	23.4 ± 0.6	2247.8 ± 48.2	47.1 ± 2.9
RG (*n* = 11)	3 F, 8 M	24.6 ± 1.6	25.5 ± 2.1	2363.6 ± 96.6	44.7 ± 7.8
WG (*n* = 35)	22 F, 13 M	26.2 ± 1	22.8 ± 0.5	2211.4 ± 55	47.9 ± 3

^1^ Values are means ± standard error of the mean (SEM).

**Table 3:** Products contained in the market baskets. For reference the 2000 kcal portions are provided. All products were packaged by the metabolic kitchen, and no original labels or brand names were attached to the products given to the subjects.

Consumable	Refined Grain Products		Whole Grain Products		Servings/Week
Food Item	Description	Brand	Description	Brand	At 2000 kcal
Wheat bread	White, slices	Sysco Classic	100% whole wheat, slices	Hi Vibe	7 slices
Cereal	Cornflakes	Kellogg’s	Wheaties	General Mills	5 cups
Cookie	Chocolate chip with white enriched flour	Recipe developed for study	Chocolate chip with whole wheat flour	Recipe developed for study	2 cookies, 2 ½ inch diameter
Couscous	Refined Wheat	Giusto	Whole Wheat	Woodland Farms	½ cup prepared + 3 oz. dry
Crackers	Goldfish, cheddar, original	Pepperidge Farms	Goldfish, cheddar, whole wheat	Pepperidge Farms	26 crackers
Corn Muffin	Made with finely ground cornmeal	Recipe developed for study	Made with whole kernel cornmeal	Recipe developed for study	2 muffins
Penne Pasta	Semolina Wheat	La Bella	Semolina Whole Wheat	La Bella	½ cup prepared + 2 oz. dry
Rice	Long-rain, white	Sysco Classic	Long-grain, brown	Sysco Classic	½ cup prepared + 4 oz. dry
Spaghetti	Semolina Wheat	La Bella	Semolina, Whole Wheat	La Bella	½ cup prepared + 2 oz. dry
Tortilla	Wheat	Mi Rancho	Whole Wheat	Mi Rancho	1 tortilla (12-inch diameter)
Baking Mix	Based on enriched white flour	Formula developed for study	Based on whole wheat flour	Formula developed for study	1 cup

**Table 4:** Change in body mass index and body composition over the six-week intervention for refined grain (RG) and whole grain (WG) and treatments ^1,2^.

Treatment	Change in BMI (kg/m^2^)	Change in Fat Mass (kg)	Change in Fat Free Mass (kg)
RG	0.01 ± 0.11	1.13 ± 0.57	−0.45 ± 0.56
WG	0.05 ± 0.10	0.49 ± 0.31	−0.43 ± 0.37
*p* value	0.846	0.936	0.936

^1^ Values are reported as the mean ± the standard error of the mean; ^2^ Change in BMI (RG: *n* = 11, WG: *n* = 34), change in Fat Mass and Fat Free Mass (RG: *n* = 10, WG: *n* = 33).

**Table 5:** GI symptoms reported at Weeks 1 and 6 of the refined grain (RG) (*n* = 10) and whole grain (WG) (*n* = 27) interventions ^1,2^.

Treatment	Bloating and Gas	Abdominal Pain	Nausea	Flatulence	Change in Stool	Feeling about Change in Stool	Bowel Movement Frequency	Average Bristol Score
Week 1 Responses								
RG	0.80 ± 0.29	0.70 ± 0.30	0.60 ± 0.31	0.90 ± 0.38	0.8 ± 0.25	2.14 ± 0.25	7.00 ± 0.61	3.18 ± 0.23
WG	0.78 ± 0.20	0.56 ± 0.12	0.38 ± 0.12	1.04 ± 0.24	1.17 ± 0.21	2.14 ± 0.23	8.28 ± 0.65	4.16 ± 0.54
*p* value	0.824	0.863	0.595	0.573	0.371	0.785	0.397	0.088
Week 6 Responses								
RG	0.40 ± 0.22	0.20 ± 0.13	0.10 ± 0.10	0.60 ± 0.31	0.90 ± 0.28	2.00± 0.19	7.00 ± 0.70	3.42 ± 0.25
WG	0.64 ± 0.14	0.44 ± 0.10	0.40 ± 0.14	0.68 ± 0.18	0.92 ± 0.20	2.18 ± 0.23	7.93 ± 0.59	3.41 ± 0.14
*p* value	0.307	0.198	0.187	0.774	0.952	0.518	0.535	0.959

^1^ Values are means ± SEM. ^2^ Higher values represent increases in symptoms, frequency, changes, positive feelings, or firmness of feces while lower numbers represent decreases in symptoms, frequency, changes, positive feelings, or firmness of feces.
